# The relative importance of education and health behaviour for health and wellbeing

**DOI:** 10.1186/s12889-023-16943-7

**Published:** 2023-10-11

**Authors:** Jan Abel Olsen, Gang Chen, Admassu N. Lamu

**Affiliations:** 1 Department of Community Medicine, UiT - the Arctic University of Norway, Tromsø, Norway; 2 Centre for Health Economics, Monash University, Melbourne, Australia; 3 Division for Health Services, Norwegian Institute of Public Health, Oslo, Norway; 4 NORCE- Norwegian Research Centre, Bergen, Norway

**Keywords:** Educational attainment, Health behaviours, Healthy lifestyle index, Childhood circumstances, Inequalites, EQ-5D, Subjective wellbeing, Norway, The Tromsø Study

## Abstract

**Background:**

Indicators of socioeconomic position (SEP) and health behaviours (HB) are widely used predictors of health variations. Their relative importance is hard to establish, because HB takes a mediating role in the link between SEP and health. We aim to provide new knowledge on how SEP and HB are related to health and wellbeing.

**Methods:**

The analysis considered 14,713 Norwegians aged 40–63. Separate regressions were performed using two outcomes for health-related quality of life (EQ-5D-5 L; EQ-VAS), and one for subjective wellbeing (Satisfaction with Life Scale). As predictors, we used educational attainment and a composite measure of HB – both categorized into four levels. We adjusted for differences in childhood financial circumstances, sex and age. We estimated the percentage share of each predictor in total explained variation, and the relative contributions of HB in the education-health association.

**Results:**

The reference case model, excluding HB, suggests consistent stepwise education gradients in health-related quality of life. The gap between the lowest and highest education was 0.042 on the EQ-5D-5 L, and 0.062 on the EQ-VAS. When including HB, the education effects were much attenuated, making HB take the lion share of the explained health variance. HB contributes 29% of the education-health gradient when health is measured by EQ-5D-5 L, and 40% when measured by EQ-VAS. For subjective wellbeing, we observed a strong HB-gradient, but no education gradient.

**Conclusion:**

In the institutional context of a rich egalitarian country, variations in health and wellbeing are to a larger extent explained by health behaviours than educational attainment.

**Supplementary Information:**

The online version contains supplementary material available at 10.1186/s12889-023-16943-7.

## Introduction

The strong association between individuals’ socioeconomic position (SEP) and their health is well documented: the social gradient is ubiquitous and persistent. This is evidenced even in the Nordic countries, despite their generous social insurance schemes and low level of income inequalities, a finding referred to as *the Nordic paradox* [1]. A large body of literature has emerged to explain the causal mechanisms, or the social determinants of health inequalities [2, 3].

Another stream of literature seeks to explain health disparities by differences in health behaviours (HB). Most deaths are now caused by non-communicable chronic diseases, often referred to as ‘lifestyle diseases’ due to their links to lifestyle behaviours [4]. Given the observed association between SEP and HB, the social gradient in health would to some extent reflect an underlying social gradient in health *behaviours* [5]. A comprehensive review suggested that HB contributes 24% of the SEP-health gradient in all-cause mortality, and that this contribution was higher in North America and Northern Europe [6].

In this vast literature, alternative indicators are being used to measure each of these key variables: SEP is commonly measured by education, income or occupation; HB is commonly measured by a single lifestyle factor such as smoking, alcohol, physical activity *or* BMI; and health is measured by a wide range of outcomes related to life expectancy, morbidities or quality of life. In the current study, we chose the most widely used SEP-indicator, namely educational attainment, while, for (un)healthy behaviours, we chose to develop a composite indicator that integrates the four abovementioned lifestyle factors. To test the sensitivity of the results, we apply two different outcome measures for health-related quality of life (HRQoL): EQ-5D-5 L and EQ-VAS [7].

Furthermore, we extend the scope of the analysis beyond health, by investigating the relative importance of SEP vs. HB on subjective wellbeing (SWB), as measured by the Satisfaction with Life Scale (SWLS), one of the most widely used subjective wellbeing (SWB) measures globally [8]. Recent studies from diverse institutional settings have shown consistent positive associations between HB and SWB [9, 10]. However, as for the association between educational attainment and SWB, the evidence is scarce, inconsistent, and reports of a negative association are common [11]. Thus, given the increased policy attention on how to improve people’s wellbeing, there is a need for more knowledge on the combined associations of education and HB on SWB.

Set in the institutional context of a rich egalitarian country, and based on a sample of 15,000 middle-aged Norwegian adults, we aim to provide new insights on two key questions: (i) What is the relative importance of SEP and HB for the explained variations in health and wellbeing?; (ii) How much is the contributions of HB in the SEP-health/wellbeing gradients?

The paper makes several contributions. First, due to an inherent problem in the use of educational attainment as the SEP-indicator in samples with huge discrepancies between old and young cohorts in their years of schooling, we consider a narrow age range who are old enough to have completed their education, and young enough to have been affected by the same important education reforms. Second, we applied a composite HB that integrates four key lifestyle factors. Third, we acknowledge the problem of reverse causality, and test the models on a sample that excluded subjects who had reported severe problems in their functioning, something which affect their capability for physical activity. Fourth, we applied three alternative outcome measures for health and wellbeing. Lastly, we adjust for differences in childhood financial circumstances (CFC), reflecting the evidence that early life circumstances may have lasting effects on adult health and wellbeing.

## Methods

### Data

We use data from the latest wave of an ongoing population-based health study in the largest city in Northern Norway; The Tromsø Study, conducted in 2015/16 (N = 21,083) that includes adults aged 40 and above [12]. With a wide age range [40–93], the distribution across educational attainment levels differs immensely between the youngest and the oldest cohort. For the current paper, we exclude participants born *before* 1952, because they were not exposed to policy reforms that had substantial effects on the uptake of higher education (see footnote to Table 1). Thus, we narrow the age range to [40–63] (N = 14,713). The study is approved by the Regional Committee for Medical and Health Research Ethics (ID 2016/607).

**Table 1:** Sample characteristics

Variables	N	Mean (SD)/%
Education, n (%)		
Primary	2313	15.9
Secondary	4182	28.7
Tertiary low	3063	21.0
Tertiary high	5029	34.5
Health behaviour, n (%)		
Super-healthy^a^	1938	13.5
Semi-healthy^b & c^	2363	16.5
Semi-unhealthy^d^	6667	46.4
Unhealthy^e & f^	3401	23.7
Childhood financial circumstances (CFC), n (%)		
Difficult	3279	22.7
Good	11,183	77.3
Sex, n (%)		
Female	7818	53.1
Male	6895	46.9
Age [40–63]^g^	14,713	51.2 (6.8)
Health, mean (SD)		
EQ-5D-5 L index	14,226	0.89 (0.11)
EQ-VAS score	14,503	0.77 (0.16)
Wellbeing, mean (SD)		
SWLS-3 value	14,274	0.70 (0.21)

^a^ Non-smokers; alcohol ≤ 14 units per week; BMI [18.5–25]; physical activity (PA) ≥ 150 min per week

^b^ Non-smokers; alcohol ≤ 14 units per week; BMI [18.5–25]; PA [60–149] minutes per week

^c^ Non-smokers; alcohol ≤ 14 units per week; BMI [25–27.49]; PA ≥ 150 min per week

^d^ All residual combinations other than defined by sub-groups a, b, c, e, f

^e^ BMI > 30 & PA < 60 min per week & non-smokers

^f^ All smokers, no matter their BMI, PA or alcohol intake

*EQ-5D-5 L*: EuroQol descriptive system, using the WePP value set (Western Preference Pattern, hybrid based on four Western countries: Canada, England, the Netherlands, Spain);

*EQ- VAS*: EuroQol Visual Analogue Scale, converted to [0–1] scale;

*SWLS-3*: the first three items of the satisfaction with life scale, converted to [0–1] scale

^g^ The 63-year-old cohort was the first in this municipality to be exposed to an educational reform that extended primary school from 7 to 9 years of compulsory education, something which appears to have contributed to a generally higher education level, in that the proportion of tertiary educated increased from 42.0% among the 64 year old subjects to 45.8% among the 63-year-olds. A further reform that affected higher uptake of tertiary education was the expanded financial support for education around the time when the 63-year-olds finished secondary school

### Outcome variables

HRQoL was measured by the EQ-5D-5 L index, as well as the EQ-VAS. The EQ-5D-5 L is a generic preference-based descriptive system that includes five dimensions: mobility, self-care, usual activities, pain/discomfort, and anxiety/depression, each with five severity levels (*no problems*, *slight problems*, *moderate problems*, *severe problems*, or *unable to/extreme problems*) [7]. In the absence of a Norwegian value set, we applied an amalgam value set based on four Western countries’ preference pattern (WePP) [13]. The EQ-5D-5 L index is anchored on a [0 (death) to 1 (full health)] scale. The EQ-VAS score is based on respondents’ direct valuations of their overall health on a visual analogue scale that ranges from 0 (*worst imaginable health*) to 100 (*best imaginable health*). We rescaled the EQ-VAS scores to [0–1].

SWB is assessed by SWLS [14]. We use the first three items, referred to as SWLS-3: *In most ways my life is close to my ideal; The conditions of my life are excellent; I am satisfied with my life.* The response options ranged from 1 (strongly disagree) to 7 (strongly agree). The total sum of score is linearly transformed onto a [0–1] scale. The omitted two items are sensitive to age as they implicate experience of life satisfaction in the past [15, 16], and they have poorer psychometric properties [17, 18].

### Predictors

The main predictors are educational attainment and HB. Education is categorized in line with the International Standard Classification of Education: (1) primary (including lower secondary); (2) secondary (including vocational); (3) tertiary low (less than 4 years of university study); (4) tertiary high (4 years or more of university study).

HB was measured by the use of a composite indicator that integrates the four lifestyle behaviours that are most often included in a ‘healthy lifestyle index’: smoking, alcohol, physical activity and BMI (see e.g. [19], [20], [21], [22]). Official public health recommendations commonly concentrate on the same four behaviours [23].

The first – do not *smoke* – is the least contentious. As to *alcohol* consumption, there is less consensus on exactly which maximum weekly intake is considered healthy, and whether the level should be lower for women than men. The Chief Medical Officers’ guidelines in the UK recommend not to drink more than 14 units a week on a regular basis, and that this level be the same for men and women [24].

The third behaviour is *physical activity*. World Health Organization (WHO) guidelines recommend at least 150 min of moderate-intensity physical activity (PA) per week (pw) [25]. The BMI is included as a fourth measure of health behaviour, expressed in units of kg/m^2^. Both height and weight were *objectively* measured in our study. A ‘normal BMI’ ranges between [18.5–25); underweight is defined as less than 18.5; ‘overweight’ is in the range [25–30); ‘obese’ is 30 kg/m^2^ and above.

The composite HB indicator is defined on four levels of (un)healthy behaviour: the *super-healthy*, the *semi-healthy*, the *unhealthy*, and a residual reference level, referred to as *semi-unhealthy*, see Table 2. The sub-group of individuals who adhere to *all four* public health recommendations is referred to as *super-healthy*, i.e. they do *not* smoke; they do not consume above 14 units of alcohol pw; they are physically active (exercise > 150 min pw); and they fall into the category of a ‘normal BMI’.

**Table 2:** Associations of education and health behaviour with health and wellbeing, adjusted for childhood financial circumstances, age and sex

	EQ-5D-5 L			EQ-VAS			SWLS-3		
Variables	Model-1	Model-2	% of Model-2 R^2^	Model-1	Model-2	% of Model-2 R^2^	Model-1	Model-2	% of Model-2 R^2^
Education (ref. Primary)			21.4			16.1			6.6
Secondary	0.017***	0.013***		0.022***	0.015***		0.009	0.003	
	(0.003)	(0.003)		(0.005)	(0.005)		(0.006)	(0.006)	
Tertiary low	0.028***	0.021***		0.038***	0.022***		0.010	-0.004	
	(0.003)	(0.003)		(0.005)	(0.005)		(0.006)	(0.006)	
Tertiary high	0.042***	0.030***		0.062***	0.037***		0.035***	0.013**	
	(0.003)	(0.003)		(0.004)	(0.004)		(0.006)	(0.006)	
Health behaviour (HB) (ref. Unhealthy)			39.5			70.3			54.9
Semi-unhealthy		0.025***			0.046***			0.057***	
		(0.003)			(0.004)			(0.005)	
Semi-healthy		0.041***			0.088***			0.077***	
		(0.003)			(0.004)			(0.006)	
Super-healthy		0.047***			0.111***			0.089***	
		(0.003)			(0.004)			(0.006)	
Childhood financial circumstances (CFC) (ref. Good)			20.4			12.7			34.3
Difficult	-0.028***	-0.027***		-0.038***	-0.035***		-0.060***	-0.057***	
	(0.002)	(0.002)		(0.003)	(0.003)		(0.004)	(0.004)	
Sex									
Male	0.023***	0.025***	18.5	0.004*	0.010***	0.4	0.002	0.005	0.2
	(0.002)	(0.002)		(0.003)	(0.003)		(0.004)	(0.004)	
Age	0.0002	0.0002	0.2	0.001***	0.001***	0.6	0.001***	0.001***	4.0
	(0.0001)	(0.0001)		(0.000)	(0.000)		(0.000)	(0.000)	
Constant	0.850***	0.832***		0.709***	0.669***		0.626***	0.585***	
	(0.008)	(0.008)		(0.011)	(0.011)		(0.015)	(0.015)	
Observations	14,031	13,835		14,304	14,103		14,086	13,904	
Overall R-squared	0.042	0.062		0.031	0.080		0.020	0.040	
Contributions of HB to Education gradient		28.6%			40.3%			62.9%	

*EQ-5D-5 L*: EuroQol descriptive system using the WePP value set (Western Preference Pattern, hybrid based on four Western countries: Canada, England, the Netherlands, Spain); *EQ- VAS*: EuroQol Visual Analogue Scale, converted to [0–1] scale; *SWLS-3*: the first three items of the Satisfaction With Life Scale, converted to [0–1] scale

*% of Model-2 R*^*2*^ was calculated using Shapley values of five variables included in Model-2. It represents the contribution of each (group of) variable to the overall R^2^ in Model-2. Take explaining EQ-5D-5 L as an example, HB contributed to 39.5% of the explained variance (R^2^ = 0.062)

Robust standard errors in parentheses. *** p < 0.01, ** p < 0.05, * p < 0.1

The *semi-healthy* includes two sub-groups who do not fully satisfy *either* of the PA *or* the BMI requirements. The first represents individuals who are only *moderately physically active* (60–150 min pw), but otherwise healthy in that they do not smoke, have low alcohol, and a normal BMI. We relaxed the PA level because many individuals may well be physically active without perceiving their activities as *exercising*, as conveyed in the questions on which the variable used is calculated (*How often do you exercise? For how long time do you exercise on average?*). Furthermore, there is a diminishing marginal health effect of increasing PA-levels [26, 27]. The second sub-group represents individuals who are *slightly overweight* with a BMI in the range [25–27.5), but otherwise healthy in that they do not smoke, have low alcohol, and are physically active (> 150 min pw). The reasoning behind relaxing the BMI is an apparent controversy in the literature suggesting that slight overweight is not associated with shorter life expectancy [28], particularly so for men [29].

The *unhealthy* also includes two sub-groups: (i) smokers; and (ii) individuals who are obese *and* physically inactive (defined as less than 60 min pw). While there is a wide consensus that smoking is unhealthy, there is less consensus on exactly how unhealthy inactivity or obesity are. Still, there is much evidence that the *combination* of obese *and* inactive is unhealthy [26].

Besides age and sex, we adjusted for childhood financial circumstances (CFC), which is evidenced to have lasting impacts on adult health and wellbeing [30–32]. CFC is measured by the question: *How was your family’s financial situation during childhood?* The response options *Very good* and *Good* were merged into a reference category Good, while response options *Difficult* and *Very difficult* were merged into Difficult. Similar indicators have been used to proxy childhood socio-economic circumstances in a range of epidemiological studies [33, 34].

### Statistical analyses

In the interest of providing a simple background figure, we estimate mean health (EQ-5D-5 L; EQ-VAS) and wellbeing (SWLS-3) by educational attainment and level of healthy behaviour.

Two regression models are estimated separately on the three outcome measures. Model-1 represents the simple reference containing education only, controlling for age, sex and CFC. Model-2 further includes the HB variable. Furthermore, by use of Shapley value decompositions, we estimate the percentage share of each variable in total explained variation (R^2^) in Model-2 for each of the three measures of outcomes. We also tested for interaction terms, i.e., whether the effects of health behaviour on HRQoL and wellbeing depends on the level of educational attainment. Based on the full model (Model-2), the results from likelihood ratio test revealed statistically insignificant interaction effects. The likelihood ratio tests for our three outcome variables are: EQ-5D-5 L [chi-squared with 9 degrees of freedom (${\chi }_{\left(9\right)}^{2}$) = 6.89, p = 0.648]; VAS [${\chi }_{\left(9\right)}^{2}$ = 8.45, p = 0.489]; and SWB [${\chi }_{\left(9\right)}^{2}$ = 9.11, p = 0.427].

To be consistent with Petrovic, de Mestral [6], we estimated the relative contributions of HB to the education gradient in each of the three outcome measures by comparing the magnitude of the education coefficients across the two models using the simple formula adopted:

Contribution of HB (%) = 100 * (β_Model−1_ –β_Model−2_)/β_Model−1_.

Where β refers to the estimated β coefficient of the highest educational attainment (i.e. Tertiary high). This simple calculation enables direct comparisons with the literature [6], yet it omits the potential nonlinear influence of HB for different educational attainments.

As a sensitivity analyses the same regression models were run for Model-1 and Model-2 on a sample that excluded the 681 respondents (4.6%) who had reported severe and/or extreme problems on at least one of the first four dimensions (on functioning and pain) in the EQ-5D-5 L. Thus, respondents with these health profiles were excluded on grounds that a non-healthy behaviour might be caused by their initially ill-health, i.e., reflect they are *unable* rather than *unwilling* to make healthy efforts. Assuming that their lower HRQoL (outcome variable) would impact on their capacity for physical activity (predictor variable), we investigated the robustness of our results on a reduced sample of ‘non-ill-health’ subjects. We expect the magnitude of the key coefficients to be reduced in these estimations, because a generally healthier sample is being considered.

## Results

Table 1 shows that similar to the national statistics, participants in the Tromsø Study are highly educated, with around 50% who have tertiary attainment. A closer investigation of the *semi-unhealthy* group showed that the most frequent combination was *slight overweight & moderately active & low alcohol* (n = 916, see also footnote under Table 2).

**Fig. 1:**
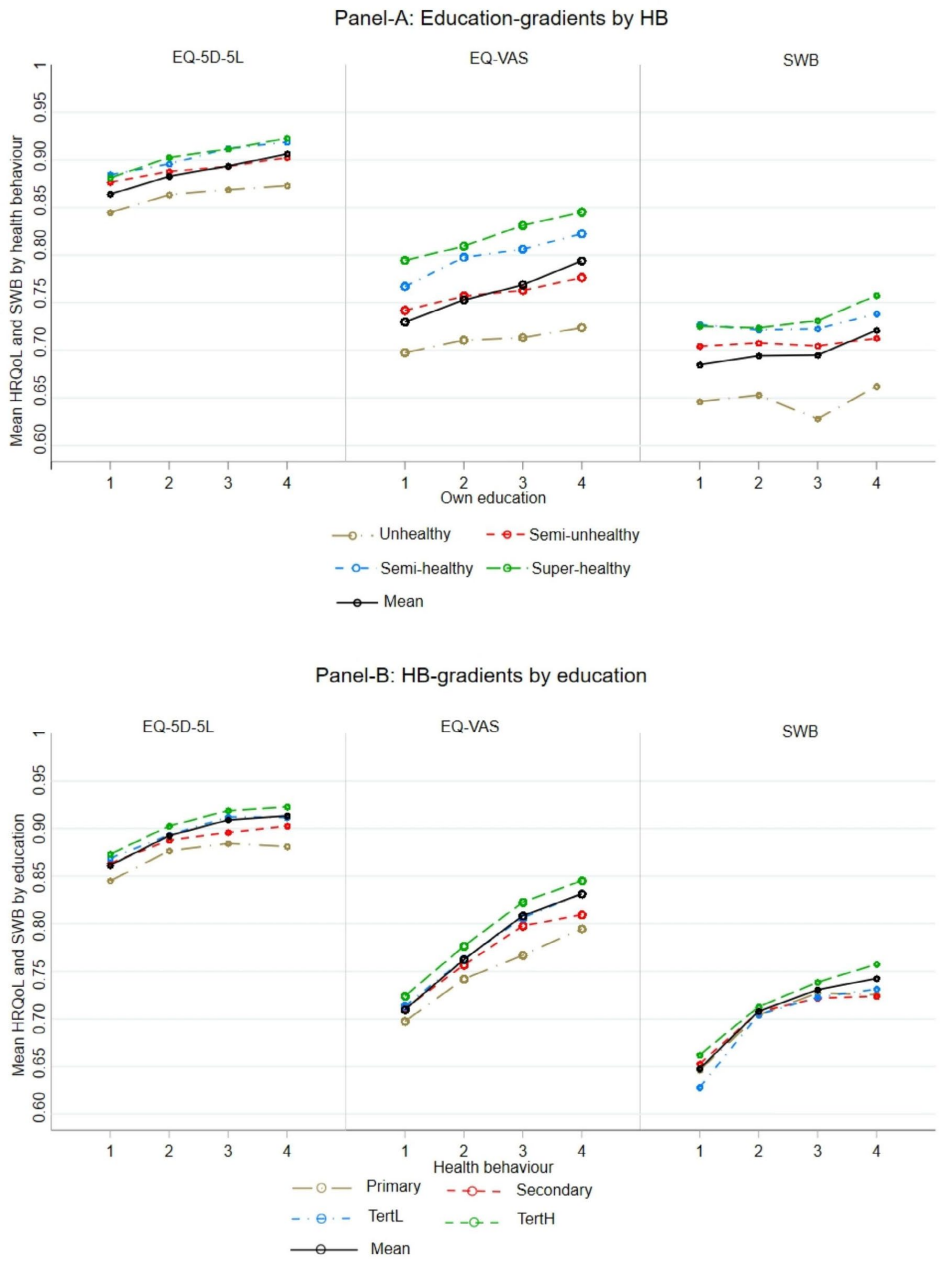
Mean HRQoL and SWB by educational attainment and level of healthy behaviour. Panel-A is education-gradients in HRQoL and SWB by each level HB; Panel-B is HB-gradients in HRQoL and SWB by each level education. *EQ-5D-5 L*: EuroQol five-dimensional five-level; *EQ-VAS*: Visual analogue scale; *SWB*: Subjective wellbeing; *HRQoL*: Health-relateed-quality of life (measured by EQ-5D-5 L and VAS); *HB*: Health behaviour, with 4 levels: 1 = Unhealthy; 2 = Semi-unhealthy; 3 = Semi-healthy; 4 = Super-healthy). Education, with 4 levels: 1 = Primary; 2 = Secondary; 3 = Tertiary low; 4 = Tertiary high

Figure 1 (based on Table A1) illustrates the contrasts in health and wellbeing by educational attainment and HB. In Panel A, we observe a consistent education gradient for both HRQoL measures along each level of HB, i.e. within each of the four HB-levels, the higher your education, the better your health. In contrast to the two health outcomes, there is no indication of an education gradient in SWB.

Panel B illustrates the HB-gradients along each level of education. We observe clear gradients along each of the four education levels, on all three measures of outcomes. Note also the clear pattern of steeper gradients in Panel B than in Panel A; suggesting consistently stronger associations between HB and the three outcomes, than between education and the outcomes.

Table 3 presents our main regression results. Model-1 confirms previous literature showing a consistent education-health gradient, i.e. for each increased level of education, we observe better HRQoL, both for EQ-5D-5 L and EQ-VAS. However, there is no corresponding education-wellbeing gradient, except for the highest education level, which is positively significant.

**Table 3:** Categorizing combinations of four health behaviours into four levels of (un)healthy behaviours

Super-healthy	Semi-healthy		Semi-unhealthy*	Unhealthy	
Adhere to public health recommendations	Moderate PA	Slight overweight	Residual combinations	Obese and inactive	Smokers
$\checkmark$ Non-smoker $\checkmark$ Alcohol ≤ 14 units pw $\checkmark$ BMI [18.5–25] $\checkmark$ PA ≥ 150 min pw	$\checkmark$ Non-smoker $\checkmark$ Alcohol ≤ 14 units pw $\checkmark$ BMI [18.5–25] $\checkmark$ PA 60–149 min pw	$\checkmark$ Non-smoker $\checkmark$ Alcohol ≤ 14 units pw $\checkmark$ BMI [25–27.49] $\checkmark$ PA ≥ 150 min pw	All other combinations than those defined by the other sub-groups	$\checkmark$ Non-smoker $\checkmark$ *Alcohol – any* $\checkmark$ BMI > 30 $\checkmark$ PA < 60 min pw	$\checkmark$ Current smoker $\checkmark$ *Alcohol – any* $\checkmark$ *BMI – any* $\checkmark$ *PA – any*

*The five most frequent combinations accounted for nearly two thirds (4141/6667) of the total Semi-unhealthy group, all five with low alcohol consumption: Slight overweight & Moderate PA; Obese & Moderate PA; Obese & Active; Normal BMI & Inactive; and BMI [27.5–30) & Moderate PA

In Model-2, strong and consistent effects of HB emerge on all three outcome measures: significant improvements in health and wellbeing for each HB-level. While education effects are attenuated, there are only minor changes to the coefficients of CFC, sex and age.

The Shapley value decomposition (%R^2^) shows the relative importance of each predictor in the overall explained variance (R^2^) of health and wellbeing in Model-2. Generally, HB explains much more than education. Lastly, the bottom row in Table 3 shows the contribution of HB in the education gradient in health and wellbeing associated with the highest vs. the lowest education, which differs across the three outcomes: 28.6% in EQ-5D-5 L; 40.3% in EQ-VAS; and 62.9% in SWB.

Results from a reduced sample of ‘non-ill-health’ subjects were reported in Supplementary material, Table A2. The same general pattern of results was observed, however, with attenuated coefficients. As compared to the full sample, in this reduced sample, the contribution of HB to the education gradient was somewhat lower in the EQ-5D-5 L model, but similar for EQ-VAS and SWLS-3.

## Discussion

In the context of a rich egalitarian country, we have provided new evidence to show that the education-health gradient persists, albeit weakened, when differences in health behaviours have been accounted for. Our study suggests that health behaviour contributes 29–40% of the education-health gradient in Norway, depending on which HRQoL outcome is used (EQ-5D-5 L or EQ-VAS). These results are larger than the median figures reported in the extensive recent review [6], however, our results might reflect that relative contributions were found to be larger in Northern Europe. Another reason might be the different outcomes used: we have considered HRQoL outcomes, while the review included all-cause mortality, CVD and cancer. However, mortality (or *quantity* of life) is strongly associated with health-related *quality* of life (HRQoL), and so are severe morbidities. A recent systematic review of socioeconomic inequalities in health also suggests that, among others, education and health behaviours are factors that appear to be associated with low HRQoL across Europe [35].

As for the relative importance of education vs. HB, our results suggest that HB is the most important for the explained variance in health. According to the Shapley value decompositions, HB was twice as important as educational attainment for the explained variance in EQ-5D-5 L, and contributes the lion’s share (70%) in EQ-VAS.

When considering subjective wellbeing as the outcome, our results suggest consistent increases in SWB along each of the four levels of HB. These findings correspond with recent studies from Finland, US and Japan [9, 10, 36], suggesting there exists bi-directional relationships between healthy behaviour and life satisfaction. However, we found no indication of an education-wellbeing gradient, apart from the highest education level reporting slightly higher SWB. This absence of an association between educational attainment and SWB should not be surprising, given the very mixed evidence in recent literature [11, 37, 38]. For example, respondents with higher educational attainment could have higher expectations which are not necessarily realized. Meanwhile, the associations could vary by different life domains whilst, in this study, we only considered global life satisfaction.

Lastly, the confounding effects of deprived childhood circumstances on adult health point in the expected direction. Interestingly, when comparing the coefficients in Model-1 and Model-2, this effect remains stable, i.e. we observed strong significant associations between CFC and health, independent of HB and educational attainment. Sadly, for those who have been unlucky in their early life lottery, our results suggest that deprived childhood circumstances have even stronger negative effects on wellbeing than on health.

There are some limitations to this study. While this study clearly defines (un)healthy behaviours by combining the four most widely used lifestyle indicators, the computation of our composite HB can be challenged as crude since we included only four levels. On the spectrum between super-healthy and unhealthy, there are numerous combinations that lean towards being quite healthy or quite unhealthy. However, it was beyond the scope of the current paper to develop a healthy lifestyle index as measured by the expected life-year losses associated with all the 60 possible lifestyle combinations (i.e. 2 smoking levels * 2 alcohol levels * 3 PA-levels * 5 BMI levels). Rather, we chose to concentrate on those combinations that are clearly unhealthy or healthy. More research is needed to create a fine-grained composite measure of (un)healthy behaviour, whereby weights reflect the expected disease burden that results from various lifestyle combinations. Secondly, to enable a direct comparison with the recent review [6], instead of conducting a mediation analysis, we adopted a simple calculation focusing on the tertiary education level to understand the effect of HB on explaining the education gradient in health and wellbeing.

Finally, as always with cross-sectional data, our study explores associations, and could not claim causality. A general problem when using cross-sectional data to study the hypothesized positive effects of healthy behaviour on health outcomes is that of *reverse causality* [39]. For example, there is no way to tell whether study members who observe a healthy lifestyle did not have a previous change in their quality of life which motivated the behavioral change. However, in an attempt to reduce this problem, we rerun the full regression models on a sample where we exclude respondents whose health profile suggest they might be less capable to undertake physical activity, i.e., those who had reported severe and/or extreme problems on at least one of the first four dimensions of the EQ-5D-5 L. The findings are generally consistent with the main results from the full sample, implying that educated individuals with healthy lifestyle are healthier and happier compared to less educated people with poor lifestyle. It lies beyond the scope of the current paper to include theoretical discussions of the relationship between health behaviours and quality of life or any other measure of wellbeing (see e.g., [40, 41]). More research is needed to provide insights into these social and psychological pathways, preferably by use of longitudinal data.

### Electronic supplementary material

Below is the link to the electronic supplementary material.

Supplementary Material 1

## Data Availability

The datasets generated and/or analysed during the current study are not publicly available due licencing arrangement. Data are however available from the corresponding author upon reasonable request and with permission of scientific board of The Tromsø Study, UiT – The Arctic University of Norway, Tromsø, Norway.
