# Supplementary material for: The relative importance of education and health behaviour for health and wellbeing
Source: BMC Public Health. 2023 Oct 11;23:1981. doi: 10.1186/s12889-023-16943-7 (PMC10568892; doi:10.1186/s12889-023-16943-7)
Supplement: Supplementary file 1 — Supplementary Material 1 [file 12889_2023_16943_MOESM1_ESM.docx]

25.09.2023 Appendix

## The relative importance of education and health behaviour for health and wellbeing

**Table A1**. Mean health and wellbeing by educational attainment and level of healthy behaviour

|  | Unhealthy | | Semi-unhealthy | | Semi-healthy | | Super-healthy | | Total | |
| --- | --- | --- | --- | --- | --- | --- | --- | --- | --- | --- |
| Education | n | Mean (SD) | n | Mean (SD) | n | Mean (SD) | n | Mean (SD) | n | Mean (SD) |
| ***EQ-5D-5L*** |  |  |  |  |  |  |  |  |  |  |
| Primary | 859 | 0.845 (0.136) | 919 | 0.877 (0.108) | 222 | 0.884 (0.108) | 154 | 0.881 (0.105) | 2213 | 0.864 (0.122) |
| Secondary | 1192 | 0.863 (0.124) | 1918 | 0.888 (0.102) | 504 | 0.896 (0.094) | 369 | 0.903 (0.093) | 4035 | 0.883 (0.108) |
| Tertiary low | 611 | 0.869 (0.123) | 1461 | 0.893 (0.106) | 497 | 0.912 (0.084) | 380 | 0.912 (0.091) | 2981 | 0.894 (0.106) |
| Tertiary high | 611 | 0.873 (0.130) | 2167 | 0.903 (0.097) | 1074 | 0.919 (0.081) | 980 | 0.923 (0.086) | 4889 | 0.906 (0.099) |
| Total | 3273 | 0.861 (0.128) | 6465 | 0.892 (0.103) | 2297 | 0.909 (0.088) | 1883 | 0.913 (0.091) | 14118 | 0.890 (0.108) |
| ***EQ-VAS*** |  |  |  |  |  |  |  |  |  |  |
| Primary | 868 | 0.698 (0.181) | 942 | 0.742 (0.171) | 222 | 0.767 (0.170) | 155 | 0.795 (0.156) | 2246 | 0.730 (0.177) |
| Secondary | 1214 | 0.711 (0.174) | 1963 | 0.757 (0.155) | 514 | 0.798 (0.138) | 375 | 0.810 (0.150) | 4120 | 0.753 (0.162) |
| Tertiary low | 626 | 0.713 (0.167) | 1477 | 0.763 (0.151) | 509 | 0.806 (0.145) | 386 | 0.831 (0.132) | 3031 | 0.769 (0.156) |
| Tertiary high | 623 | 0.724 (0.162) | 2209 | 0.776 (0.142) | 1097 | 0.823 (0.129) | 1006 | 0.845 (0.123) | 4993 | 0.794 (0.144) |
| Total | 3331 | 0.710 (0.173) | 6591 | 0.763 (0.153) | 2342 | 0.808 (0.140) | 1922 | 0.831 (0.135) | 14390 | 0.767 (0.159) |
| ***SWLS-3*** |  |  |  |  |  |  |  |  |  |  |
| Primary | 831 | 0.646 (0.248) | 907 | 0.704 (0.219) | 220 | 0.727 (0.215) | 150 | 0.726 (0.202) | 2159 | 0.685 (0.231) |
| Secondary | 1190 | 0.653 (0.228) | 1932 | 0.708 (0.205) | 510 | 0.722 (0.196) | 370 | 0.724 (0.204) | 4049 | 0.694 (0.212) |
| Tertiary low | 615 | 0.628 (0.225) | 1466 | 0.705 (0.195) | 507 | 0.723 (0.187) | 384 | 0.731 (0.194) | 3004 | 0.695 (0.204) |
| Tertiary high | 618 | 0.662 (0.210) | 2193 | 0.713 (0.196) | 1090 | 0.739 (0.192) | 999 | 0.758 (0.187) | 4955 | 0.721 (0.197) |
| Total | 3254 | 0.648 (0.230) | 6498 | 0.708 (0.202) | 2327 | 0.730 (0.194) | 1903 | 0.743 (0.194) | 14167 | 0.703 (0.209) |

*EQ-5D-5L:* EuroQol descriptive system using the WePP value set (Western Preference Pattern, hybrid based on four Western countries: Canada, England, the Netherlands, Spain); *EQ- VAS*: EuroQol Visual Analogue Scale, converted to [0 – 1] scale; *SWLS-3:* the first three items of the Satisfaction With Life Scale (SWLS), converted to [0 – 1] scale.

**Appendix Table A2.** Associations of education and health behaviour with health and wellbeing among *non-ill health*^a^ respondents

|  | EQ-5D-5L | | EQ-VAS | | SWLS-3 | |
| --- | --- | --- | --- | --- | --- | --- |
| Variables | Model-1 | Model-2 | Model-1 | Model-2 | Model-1 | Model-2 |
| Education |  |  |  |  |  |  |
| Secondary | 0.011*** | 0.009*** | 0.019*** | 0.013*** | 0.003 | -0.002 |
|  | (0.002) | (0.002) | (0.004) | (0.004) | (0.006) | (0.006) |
| Tertiary low | 0.020*** | 0.016*** | 0.035*** | 0.021*** | 0.004 | -0.007 |
|  | (0.002) | (0.002) | (0.005) | (0.005) | (0.006) | (0.006) |
| Tertiary high | 0.031*** | 0.024*** | 0.055*** | 0.033*** | 0.026*** | 0.009 |
|  | (0.002) | (0.002) | (0.004) | (0.004) | (0.006) | (0.006) |
| Health behaviour |  |  |  |  |  |  |
| Semi-unhealthy |  | 0.015*** |  | 0.042*** |  | 0.050*** |
|  |  | (0.002) |  | (0.003) |  | (0.005) |
| Semi-healthy |  | 0.024*** |  | 0.076*** |  | 0.065*** |
|  |  | (0.002) |  | (0.004) |  | (0.006) |
| Super-healthy |  | 0.031*** |  | 0.100*** |  | 0.079*** |
|  |  | (0.002) |  | (0.004) |  | (0.006) |
| CFC |  |  |  |  |  |  |
| Difficult | -0.021*** | -0.020*** | -0.032*** | -0.030*** | -0.056*** | -0.054*** |
|  | (0.002) | (0.002) | (0.003) | (0.003) | (0.004) | (0.004) |
| Sex |  |  |  |  |  |  |
| Male | 0.018*** | 0.019*** | -0.002 | 0.004 | -0.003 | 0.000 |
|  | (0.001) | (0.001) | (0.003) | (0.003) | (0.004) | (0.004) |
| Age (in years) | 0.000** | 0.000* | 0.001*** | 0.001*** | 0.001*** | 0.001*** |
|  | (0.000) | (0.000) | (0.000) | (0.000) | (0.000) | (0.000) |
| Constant | 0.874*** | 0.863*** | 0.720*** | 0.685*** | 0.646*** | 0.609*** |
|  | (0.006) | (0.006) | (0.011) | (0.011) | (0.015) | (0.015) |
|  |  |  |  |  |  |  |
| Observations | 13,206 | 13,113 | 13,207 | 13,114 | 13,030 | 12,939 |
| R-squared | 0.043 | 0.058 | 0.028 | 0.073 | 0.018 | 0.034 |
| Contributions of HB to  Education gradient† | | 22.6% |  | 40.0% |  | 65.4% |

^a^This analysis is based on the non-ill-health group defined as those who described their health at level-3 or less on all the first four dimensions of EQ-5D-5L (covering functioning and pain).

*EQ-5D-5L:* EuroQol descriptive system using the WePP (Western Preference Pattern) value set; *EQ- VAS*: EuroQol Visual Analogue Scale, converted to [0 – 1] scale; *SWLS-3:* first three items of the Satisfaction With Life Scale (SWLS), converted to [0 – 1] scale.

† Contribution was calculated based on the magnitudes of the Tertiary high education coefficients across Model-1 and Model-2; that is: Contribution of HB (%) = 100 * (β_Model-1_ –β_Model-2_)/β_Model-1._

Robust standard errors in parentheses. *** p<0.01, ** p<0.05, * p<0.1
